# Prognostic Impact of Metastatic Site in Patients Receiving First-Line Sorafenib Therapy for Advanced Hepatocellular Carcinoma

**DOI:** 10.3390/cancers15051523

**Published:** 2023-02-28

**Authors:** Luca Ielasi, Francesco Tovoli, Matteo Tonnini, Bernardo Stefanini, Raffaella Tortora, Giulia Magini, Rodolfo Sacco, Tiziana Pressiani, Franco Trevisani, Ingrid Garajová, Fabio Piscaglia, Alessandro Granito

**Affiliations:** 1 Division of Internal Medicine, Hepatobiliary and Immunoallergic Diseases, IRCCS Azienda Ospedaliero-Universitaria di Bologna, 40138 Bologna, Italy; 2 Department of Medical and Surgical Sciences, University of Bologna, 40138 Bologna, Italy; 3 Liver Unit, Department of Transplantation, Cardarelli Hospital, 80131 Naples, Italy; 4 Department of Gastroenterology and Transplant Hepatology, Papa Giovanni XXIII Hospital, 24127 Bergamo, Italy; 5 Gastroenterology Unit, Azienda Ospedaliero-Universitaria Pisana, 56126 Pisa, Italy; 6 Gastroenterology and Digestive Endoscopy Unit, Foggia University Hospital, 71122 Foggia, Italy; 7 Humanitas Cancer Center, IRCCS Humanitas Research Hospital, Rozzano, 20089 Milan, Italy; 8 Semeiotica Medica, IRCCS Azienda Ospedaliero-Universitaria di Bologna, 40138 Bologna, Italy; 9 Medical Oncology Unit, University Hospital of Parma, 43126 Parma, Italy

**Keywords:** hepatocellular carcinoma, liver cancer, metastases, sorafenib, systemic therapy, radiation therapy, outcome

## Abstract

**Simple Summary:**

Several retrospective studies tried to assess the prognostic role of different sites of metastases in patients with advanced HCC, but results are often contradictory. These studies also presented results based on population samples with several confounding factors. Although the therapeutic scenario is moving towards immunotherapy, a better knowledge of a different metastatic site response rate to sorafenib is needed, also considering the potential future advent of combination therapies with immune checkpoint and tyrosine-kinase inhibitors. We tried to perform a large-scale multicentric study by enrolling metastatic HCC patients treated with sorafenib as front-line therapy. A low rate of concomitant locoregional treatments during sorafenib in our population study allowed us to focus on the actual response of different sites of metastases to systemic treatment with sorafenib, showing that lymph nodes and lung metastases have worse prognosis.

**Abstract:**

Extrahepatic spread is a well-known negative prognostic factor in patients with advanced hepatocellular carcinoma (HCC). The prognostic role of different metastatic sites and their response rate to systemic treatment is still being debated. We considered 237 metastatic HCC patients treated with sorafenib as first-line therapy in five different Italian centers from 2010 to 2020. The most common metastatic sites were lymph nodes, lungs, bone and adrenal glands. In survival analysis, the presence of dissemination to lymph nodes (OS 7.1 vs. 10.2 months; *p* = 0.007) and lungs (OS 5.9 vs. 10.2 months; *p* < 0.001) were significantly related to worse survival rates compared with all other sites. In the subgroup analysis of patients with only a single metastatic site, this prognostic effect remained statistically significant. Palliative radiation therapy on bone metastases significantly prolonged survival in this cohort of patients (OS 19.4 vs. 6.5 months; *p* < 0.001). Furthermore, patients with lymph node and lung metastases had worse disease control rates (39.4% and 30.5%, respectively) and shorter radiological progression-free survival (3.4 and 3.1 months, respectively). In conclusion, some sites of an extrahepatic spread of HCC have a prognostic impact on survival in patients treated with sorafenib; in particular, lymph nodes and lung metastases have worse prognosis and treatment response rate.

## 1. Introduction

Primary liver tumors are the sixth most common cancer and the third cancer-related cause of death worldwide with more than 900,000 new cases/year and 830,000 deaths/year. Among liver tumors, hepatocellular carcinoma (HCC) represents by far the most frequent histological type [1]. 

Despite the improvement in therapeutic strategies, the overall survival for advanced HCC patients remains poor. According to Barcelona Clinic Liver Cancer, advanced HCC (BCLC-C) is defined by the presence of macrovascular invasion (MVI), extrahepatic spread (EHS) and/or a cancer-related deterioration of general condition based on the Eastern Cooperative Oncology Group Performance Status (ECOG-PS > 0) [2].

International guidelines all agree on starting systemic therapy for advanced HCC or intermediate stage (BCLC-B) not amenable or refractory to locoregional therapies [3,4].

Since 2008, tyrosine-kinase inhibitors (TKIs) have played a central role in the pharmacologic scenario of advanced HCC. Sorafenib was the first approved drug as first-line therapy, and it represented the only therapeutic choice for these patients for about a decade [5]. In the last five years, several new TKIs have been approved for the treatment of HCC patients: lenvatinib as an alternative first-line therapy [6], regorafenib as a second-line therapy after progression to sorafenib [7], and cabozantinib as both second- and third-line therapy [8].

The presence of EHS represents an indirect sign of tumor biological aggressiveness, and its negative prognostic impact on patients’ survival has been widely demonstrated [9]. The most common metastatic sites of HCC are lymph nodes, lungs, bone and adrenal glands [10]. The prognostic role played by the different metastatic sites is less known, and their response rate to TKIs have not been yet defined in literature.

The aim of the present study is to verify whether the different metastatic sites have clinical relevance in a large multicentric population of patients treated with sorafenib for metastatic HCC.

## 2. Materials and Methods

### 2.1. Design of the Study

This study was performed using medical records from the ARPES database, a prospective multicenter registry of all consecutive HCC patients treated with sorafenib as first-line therapy. This registry was created in 2010, and it includes patients from five different Italian Centers (IRCCS Azienda Ospedaliero-Universitaria di Bologna, Bologna; Cardarelli Hospital, Naples; Papa Giovanni XXIII Hospital, Bergamo; Azienda Ospedaliero-Universitaria Pisana, Pisa; Humanitas Clinical and Research Center, Milan, Italy). Data were entered every 3–6 months by co-investigators from each center and checked by the coordinating center for internal consistency. For this study, we considered patients with radiological detection of extrahepatic HCC localizations who started sorafenib from January 2012 to December 2020. The closing follow-up date was 31 October 2022 in order to allow an adequate follow-up period.

### 2.2. Baseline and Re-Evaluation

Baseline characteristics including sex, age, laboratory findings (bilirubin, albumin, alpha-fetoprotein), liver disease characteristics (etiology, Child-Pugh score, intrahepatic tumor burden) and tumor features (MVI, EHS, ECOG-PS) were present for all patients. In all patients with EHS, the organ(s) affected by metastases was known and recorded.

In all patients, a baseline contrast-enhanced CT scan of thorax and abdomen was performed within 30 days before start of sorafenib and then every 12 ± 2 week for tumor response assessment. Treatment response from target lesions was recorded according to Response Evaluation Criteria In Solid Tumours (RECIST) v1.1 [11].

### 2.3. Management of Sorafenib

Sorafenib was started at the usual dosage of 400 mg bid. Dose modifications (reduction or temporary discontinuation) were performed in case of occurrence of intolerable adverse events. Sorafenib was permanently discontinued in case of (i) clinical and radiological progression of disease (for patients eligible for a second-line licensed drug or a clinical trial, radiological progression was considered sufficient for sorafenib interruption), (ii) unacceptable severe toxicity or intolerance and (iii) significant liver function deterioration.

According to daily clinical practice and following a multidisciplinary team discussion, concomitant or sequential locoregional treatments could have been potentially performed for better tumor burden control; such patients were also included in the database considering the real-life observational nature of the registry.

### 2.4. Statistical Analysis

Categorical variables were expressed as absolute and relative frequencies; continuous variables were expressed as mean and standard deviation. Comparisons between groups were performed using the chi-squared test for categorical variables and with Student’s *t* test for continuous variables.

Overall survival (OS) was measured from sorafenib starting date until date of patient death; last follow-up visit if no additional information could be retrieved or end of follow-up period. Radiological progression-free survival (rPFS) was measured from sorafenib starting date until date of radiological progression of disease, death or end of follow-up period. Survival curves were estimated using the Kaplan–Meier method.

Stratification of predicting factors was analyzed with Log-rank test. In order to define the independent correlation between predictive variables and survival, we performed a time-dependent covariate survival approach, including the statistically significant (*p* < 0.05) variables from the univariate Cox analysis.

Statistical analysis was performed using SPSS Statistic for MacOSX (version 24.0; IBM, Armonk, NY, USA).

## 3. Results

### 3.1. Study Population

From the entire ARPES database of 712 patients, we considered for this study only 237 patients (33.2%) with radiological detection of EHS. Most patients were male (85.2%), cirrhotic (94.1%) and with viral etiologies (69.6%). Almost all patients (95.4%) presented preserved liver function defined as Child-Pugh A; remaining patients were all in Child-Pugh score B7 liver functional class. Baseline characteristics of study population compared with other patients in ARPES registry are summarized in Table 1.

**Figure 1:**
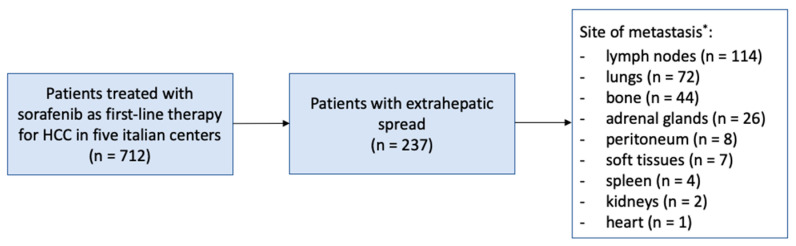
Study population and metastases localization distribution. * Multiple concomitant sites of metastases were recorded in 48 patients.

The most frequent sites of metastases were lymph nodes (48.1%), lungs (30.4%), bone (18.6%) and adrenal glands (11.0%); remaining metastatic sites presented a frequency < 5%. Multiple concomitant sites of metastases were recorded in 48 patients (20.2%) (Figure 1).

Baseline characteristics of patients with lymph node, lung, bone and adrenal gland metastases compared with other patients of the study population are reported in Table 2.

### 3.2. Survival Analysis

The univariate analysis of OS showed that compromised liver function (Child-Pugh B), high albumin-bilirubin grade (ALBI > 1), elevated serum alpha-fetoprotein values (AFP > 400 ng/mL), intrahepatic tumor burden > 50% (ITB > 50%), cancer-related deterioration of general conditions (ECOG-PS > 0), presence of microvascular invasion (MVI) and occurrence of dermatological adverse events during treatment (DAEs) were associated with patients’ prognosis.

A statistically significant independent correlation with survival was confirmed in the multivariate analysis for Child–Pugh B (HR 3.105, *p* = 0.002), AFP > 400 ng/mL (HR 1.396, *p* = 0.030), ITB > 50% (HR 1.543, *p* = 0.006), MVI (HR 1.770, *p* < 0.001) and DAEs (HR 0.755, *p* = 0.045).

**Figure 2:**
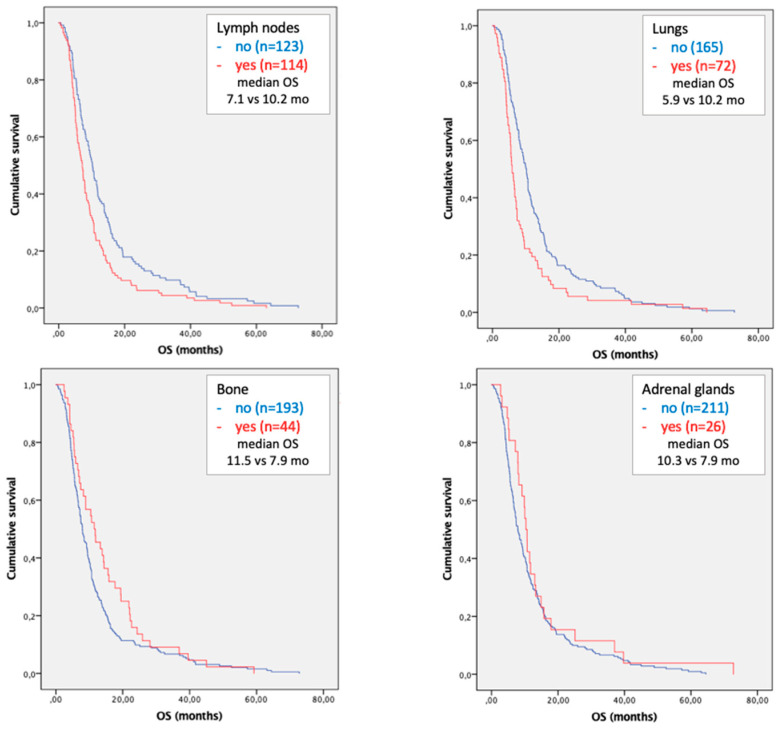
Kaplan–Meier curves of overall survival in patients with lymph node, lung, bone and adrenal gland metastases.

Regarding the sites of metastases, the presence of lymph node, lung and multiple site localizations of disease were associated with worse prognosis. A statistically independent correlation with survival was confirmed in the multivariate analysis for lymph node (HR 1.545; OS 7.1 vs. 10.2 mo; *p* = 0.007) and lung metastases (HR 2.110; OS 5.9 vs. 10.2 mo; *p* < 0.001) compared with patients with metastatic HCC without lymph node or lung localizations, respectively (Table 3 and Figure 2).

After stratification of patients with lymph node and lung metastases, patients with the co-presence of these two metastatic sites had worse survival rates compared to those who presented only lymph node (HR 2.014; OS 5.0 vs. 7.4 mo; *p* = 0.006) or only lung metastases (HR 1.762; OS 5.0 vs. 6.5 mo; *p* = 0.040).

### 3.3. Subgroup Analysis in Patients with Single Site of Metastases

In order to more accurately define the prognostic role of different site of metastases, we performed a subgroup survival analysis in patients with single metastatic site (n = 189). In this cohort of patients, Child–Pugh B (HR 5.661, *p* < 0.001), AFP > 400 ng/mL (1.464, *p* = 0.026), ITB > 50% (HR 1.614, *p* = 0.009) and presence of MVI (HR 1.770, *p* = 0.001) were significantly related to worse survivals.

**Figure 3:**
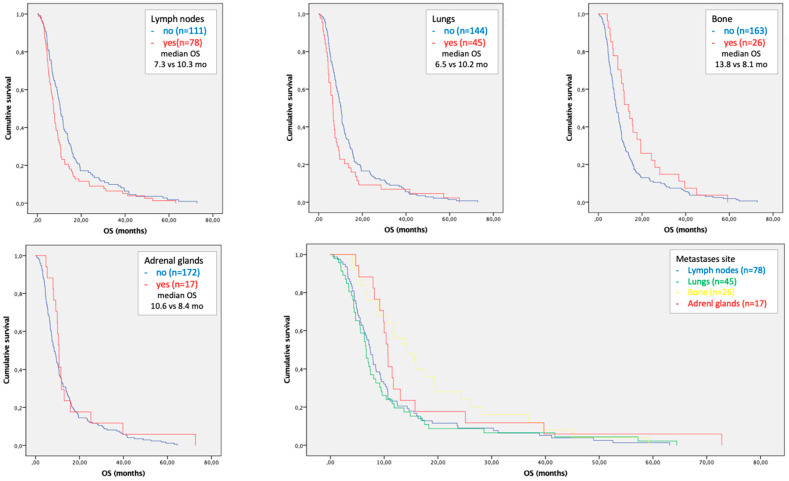
Kaplan–Meier curves of overall survival in patients with lymph node, lung, bone and adrenal gland metastases in the cohort of patients with single site of metastases. The last figure compares the survival curves of four overmentioned most frequent sites of metastases.

Regarding the sites of metastases, the presence of lymph nodes, lung and bone localizations of disease were associated with patient prognosis. In the multivariate analysis a statistically independent correlation with survival was confirmed only for lymph node (HR 1.645; OS 7.3 vs. 10.3 mo; *p* = 0.021) and lung metastases (HR 2.182; OS 6.5 vs. 10.2 mo; *p* = 0.002) compared with patients with metastatic HCC without lymph node or lung localizations, respectively (Table 4 and Figure 3).

### 3.4. Metastases Focused Combination Treatments

In order to control extrahepatic tumor burden, locoregional combination treatments were performed in 28 patients. Among these, 3 patients had lymph node metastases (all received stereotaxic radiotherapy), 2 patients had pulmonary metastases (a patient received stereotaxic radiotherapy, the other received radiofrequency ablation) and 21 patients had bone metastases (all received radiotherapy with antalgic purpose). No surgical treatments (i.e., metastasectomy) have been performed in any patients of the study population.

**Figure 4:**
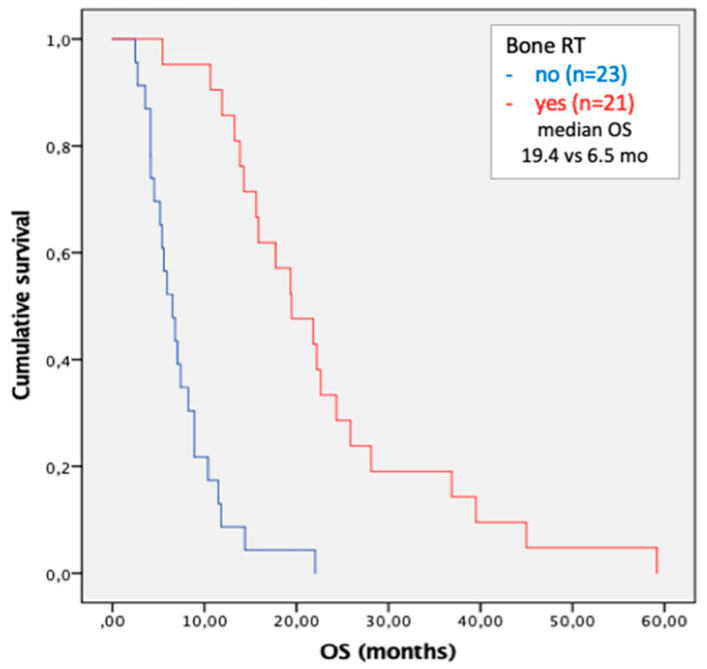
Kaplan–Meier curves of overall survival in patients with bone metastases treated with combination radiation therapy.

Due to the high number of treatments focused on bone metastases, we performed survival analysis in this cohort of patients. Radiation therapy was associated with a significant survival benefit in patients with bone metastases (HR 0.137; OS 19.4 vs. 6.5 mo; *p* < 0.001) (Figure 4).

### 3.5. Time to Progression According to Site of Metastases

A re-evaluation of all CT images was also performed in order to assess the radiological progression-free survival (rPFS) of each site of metastases. A dimensional increase of at least 20% from baseline of a target metastatic lesion or the occurrence of a new lesion in a specific site of metastases was considered as progression of disease in that metastatic site.

**Figure 5:**
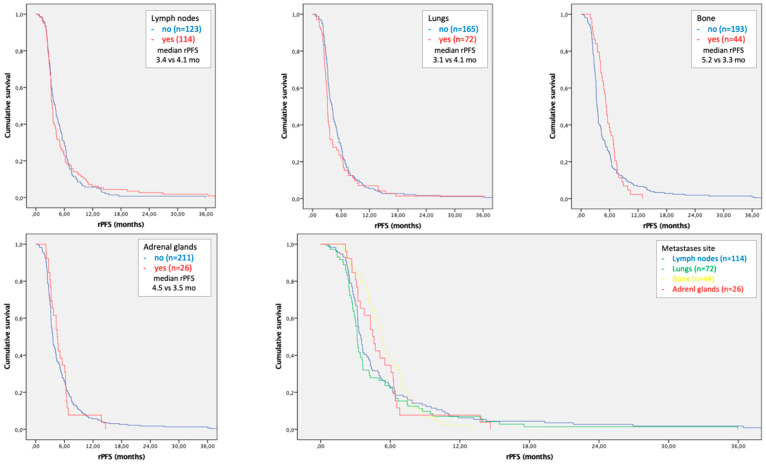
Kaplan–Meier curves of radiological progression-free survival for each site of metastases. The last figure compares the survival curves of lymph node, lung, bone and adrenal gland site of metastases.

The rPFS of lymph node, lung, bone and adrenal gland metastases was 3.4 (95% CI 3.1–3.7; *p* = 0.930), 3.1 (95% CI 2.9–3.3; *p* = 0.107), 5.2 (95% CI 4.6–5.8; *p* = 0.126) and 4.5 (95% CI 3.5–5.4; *p* = 0.719) months, respectively. No statistically significant differences in rPFS were observed among different metastatic sites (Figure 5). The disease control rates (DCRs) of the four abovementioned sites of metastases were 39.5%, 30.5%, 75.0% and 61.5%, respectively.

## 4. Discussion

Sorafenib represented the only available therapeutic option for patients with advanced HCC for several years. Since its approval, real-life studies have shown a progressive improvement in the survival of patients treated with sorafenib thanks to a better management of drug-related adverse events [12,13].

In order to better understand the wide response variability among patients, several authors investigated predictors of survival. In the last few years, several studies showed how clinical [14,15,16,17], laboratory [18,19,20,21] and pharmacologic features [22,23] could influence the prognosis of patients with advanced HCC; for instance, the presence of EHS is a well-known predictor of negative prognosis.

Regarding the actual impact of different sites of metastases on prognosis, evidence is scarce, and results are often contradictory. Two recently published works on this topic are the study of Zhan H et al., based on the American epidemiologic registry SEER (Surveillance, Epidemiology and End Results) that gives OS results [24], and the study of Huang SF et al., based on the national registry of Taiwan that gives PFS results [25]. In the first study, lymph nodes were not considered as a metastatic site, and less than a half of patients were on systemic treatment; in the second study all patients with advanced HCC were analyzed, including those without EHS.

In the study of Zhan H et al., the OS was negatively related to multiple metastatic sites, pulmonary, bone (only after propensity score matching) and lymph nodes metastases (considered as a potential influencing factor in survival analysis). In the study of Huang SF et al., the PFS was negatively related to lung and bone metastases and positively related to lymph node metastases (only in the cohort of patients with single metastatic site).

In both studies, high rates of surgical and radiation treatments were reported, but the target metastatic site was not specified.

In the current study we reported that the presence of lung or lymph node metastases are negative independent prognostic factors with a median OS of 5.9 and 7.1 months, respectively; the co-presence of these two metastatic sites reduced the median OS at 5.0 months. In the subgroup analysis of patients with a single metastatic site, a statistically significant correlation between lung and lymph node metastases and prognosis was confirmed.

Lung and lymph node metastases did not reach statistical significance in rPFS analysis even though a negative trend is represented in survival curves.

Concerning adrenal gland metastases, the fourth most common metastatic site, they did not reach statistical significance in any survival analysis, but the small sample size (26 cases) could be a determinant. Adrenal gland metastases have not been evaluated in any above-mentioned study. There are only case reports and case series on this topic in the literature, so our results on OS and rPFS (10.3 and 4.5 months, respectively) provide original, relevant information.

Combination treatments, both surgical and radiation ones, are widely used to control the tumor burden and to improve patients’ outcome [26,27]. In our study population, palliative radiation therapy on bone metastases was the most common treatment performed, so it was possible to conduct subgroup analysis in this cohort of patients. Although there was no oncologic radical purpose, antalgic radiation therapy contributed to a significant improvement in survival (median OS 19.4 vs. 6.5 months). This result could explain the positive trend observed in bone metastases OS curves. Recently, it has been demonstrated how curative radiation therapy increases survival in patients with oligometastatic (up to 5 bone lesions) solid tumors and HCC [28,29]. Nowadays, no conclusive results on the prognostic impact of palliative radiation therapy have been published [30].

In the last few years, a rapid and progressive change in the management of advanced HCC patients has occurred, leading to improved prognosis. This was due to the approval of drugs for post-sorafenib treatment [31,32] and the advent of immune checkpoint inhibitors (ICIs). Atezolizumab, a PD-L1 inhibitor, in association with bevacizumab, is the current front-line therapy for advanced HCC [33,34], and several clinical trial with ICIs, some of these in combination with TKIs, are now ongoing.

With these preconditions, TKI monotherapy seems to be outdated, but it still assumes a central role in the therapeutic scenario for patients with advanced HCC and contraindications to ICIs [35] or solid organ recipients [36]; furthermore, recent studies seem to highlight that the etiology of underling liver disease could influence the response to different first-line therapies [37].

In the next few years, the ICIs actual efficacy on the different target lesions needs to be assessed due to the close relationship between these new drugs and tumor microenvironment. The final possible aim is to tailor individual therapeutic strategies for each patient with advanced HCC.

Despite the large population of this study, the small sample size of less common sites of metastases did not allow us to perform a survival analysis in these subgroups of patients. In the current study, the enrollment of only metastatic patients in therapy with sorafenib and the low rate of concomitant treatments, with the exception of radiation therapy on bone metastases, significantly reduced possible confounders for survival analysis compared with previous studies.

## 5. Conclusions

The presence of lymph node and lung metastases are independent negative prognostic factors in patients with metastatic HCC. Radiation therapy of bone metastases, even with a palliative antalgic purpose, seems to give a survival benefit, and it should be therefore considered for the management of these patients.

## Figures and Tables

**Table 1:** Baseline characteristics of patients with extrahepatic spread (EHS) compared with all remaining patients without extrahepatic spread (all w/o EHS). The latter is reported and also divided into two groups, which were themselves split in advanced HCC without metastases (BCLC-C w/o EHS) or intermediate HCC (BCLC-B).

Variable	EHS (n = 237)	All w/o EHS (n = 475)	*p*	BCLC-C w/o EHS (n = 311)	*p*	BCLC-B (n = 164)	*p*
Male sex	202 (85.2%)	398 (83.8%)	0.637	264 (84.9%)	0.951	134 (85.2%)	0.355
Age	67.6 ± 11.5	67.8 ± 9.8	0.825	66.7 ± 10.4	0.374	69.7 ± 11.5	0.056
Viral etiology	165 (69.6%)	349 (73.5%)	0.281	231 (74.3%)	0.224	118 (69.6%)	0.630
Child-Pugh B	11 (4.6%)	47 (9.9%)	0.014	40 (12.9%)	0.001	7 (4.6%)	0.792
ALBI > 1	154 (65.0%)	381 (80.2%)	<0.001	251 (80.7%)	<0.001	130 (65.0%)	0.003
AFP > 400 ng/mL	76 (32.1%)	156 (32.8%)	0.841	116 (37.3%)	0.209	40 (32.1%)	0.115
ITB > 50%	69 (29.1%)	149 (31.4%)	0.545	106 (34.1%)	0.245	43 (29.1%)	0.628
ECOG-PS > 0	60 (25.3%)	126 (26.5%)	0.743	126 (26.5%)	0.743	N.A.	-
MVI	78 (32.9%)	232 (48.8%)	<0.001	232 (48.8%)	<0.001	N.A.	-

AFP: alfa-fetoprotein; ECOG-PS: Eastern Cooperative Oncology Group—Performance Status; ITB > 50%: intrahepatic tumor burden > 50%; MVI: macrovascular invasion. N.A. not applicable.

**Table 2:** Baseline characteristics of patients with lymph node, lung, bone and adrenal gland metastases compared with the rest of study population.

**Variable**	**Yes (n = 114)**	**Lymph Nodes** **No (n = 123)**	***p***	**Yes (n = 72)**	**Lungs** **No (n = 165)**	***p***
Male sex	93 (81.5%)	109 (88.6%)	0.108	61 (84.7%)	141 (85.4%)	0.845
Age	67.7 ± 12.5	67.6 ± 10.6	0.955	66.7 ± 9.8	68.0 ± 12.2	0.436
Viral etiology	81 (71.0%)	84 (68.2%)	0.673	46 (63.8%)	119 (72.1%)	0.062
Child-Pugh B	5 (4.3%)	6 (4.8%)	1.000	5 (6.9%)	6 (3.6%)	0.316
ALBI > 1	81 (71.0%)	73 (59.3%)	0.070	44 (61.1%)	110 (66.6%)	0.449
AFP > 400 ng/mL	36 (31.5%)	40 (32.5%)	0.890	24 (33.3%)	52 (31.5%)	0.880
ITB > 50%	33 (28.9%)	36 (29.2)	0.984	20 (27.7%)	49 (29.6%)	0.874
ECOG-PS > 0	23 (20.1%)	37 (30.0%)	0.100	20 (27.7%)	40 (24.2%)	0.627
MVI	44 (38.5%)	34 (27.6%)	0.090	25 (34.7%)	53 (32.1%)	0.764
**Variable**	**Yes (n = 44)**	**Bone** **No (n = 193)**	***p***	**Yes (n = 26)**	**Adrenal Glands** **No (n = 211)**	***p***
Male sex	41 (93.1%)	161 (83.4%)	0.155	25 (96.1%)	177 (83.8%)	0.141
Age	68.4 ± 9.6	67.4 ± 11.9	0.618	67.3 ± 10.6	67.7 ± 11.7	0.867
Viral etiology	34 (77.2%)	131 (67.8%)	0.277	20 (76.9%)	145 (68.7%)	0.134
Child-Pugh B	3 (6.8%)	8 (4.1%)	0.433	1 (3.8%)	10 (4.7%)	1.000
ALBI > 1	26 (59.0%)	128 (66.3%)	0.293	15 (57.6%)	139 (65.8%)	0.375
AFP > 400 ng/mL	16 (36.3%)	60 (31.0%)	0.592	7 (26.9%)	69 (32.7%)	0.659
ITB > 50%	10 (22.7%)	59 (30.5)	0.341	6 (23.0%)	63 (29.8%)	0.813
ECOG-PS > 0	15 (34.0%)	45 (23.3%)	0.204	5 (19.2%)	55 (26.0%)	0.633
MVI	9 (20.4%)	69 (35.7%)	0.053	7 (26.9%)	71 (33.6%)	0.659

AFP: alfa-fetoprotein; ECOG-PS: Eastern Cooperative Oncology Group—Performance Status; ITB > 50%: intrahepatic tumor burden > 50%; MVI: macrovascular invasion.

**Table 3:** Univariate and multivariate Cox regression analysis of overall survival.

Variable	Univariate HR (95% CI)	*p*	Multivariate HR (95% CI)	*p*
Male sex	1.089 (0.758–1.564)	0.644		
Viral etiology	0.918 (0.695–1.212)	0.546		
Child-Pugh B	3.615 (1.952–6.696)	<0.001	3.105 (1.538–6.268)	**0.002**
ALBI > 1	1.367 (1.038–1.801)	0.026	1.216 (0.904–1.637)	0.196
AFP > 400 ng/mL	1.478 (1.120–1.950)	0.006	1.396 (1.033–1.887)	**0.030**
ITB > 50%	1.550 (1.550–2.080)	0.003	1.543 (1.130–2.109)	**0.006**
ECOG-PS > 0	1.552 (1.152–2.092)	0.004	1.288 (0.926–1.791)	0.133
MVI	2.014 (1.522–2.080)	<0.001	1.770 (1.301–2.409)	**<0.001**
DAEs	0.759 (0.579–0.971)	0.029	0.755 (0.573–0.994)	**0.045**
Lymph nodes	1.487 (1.149–1.925)	0.003	1.545 (1.127–2.117)	**0.007**
Lungs	1.569 (1.186–2.075)	0.002	2.110 (1.484–2.999)	**<0.001**
Bone	0.759 (0.546–1.055)	0.100		
Adrenal glands	0.768 (0.506–1.164)	0.213		
Multiple sites	1.505 (1.0.91–2.075)	0.013	1.041 (0.702–1.544)	0.842

AFP: alfa-fetoprotein; DAEs: dermatologic adverse events; ECOG-PS: Eastern Cooperative Oncology Group—Performance Status; ITB > 50%: intrahepatic tumor burden > 50%; MVI: macrovascular invasion. N.A. not applicable.

**Table 4:** Univariate and multivariate Cox regression analysis of overall survival in the cohort of patients with a single site of metastases.

Variable	Univariate HR (95% CI)	*p*	Multivariate HR (95% CI)	*p*
Male sex	1.079 (0.723–1.609)	0.710		
Viral etiology	0.921 (0.672–1.262)	0.610		
Child-Pugh B	5.356 (2.577–11.131)	<0.001	6.326 (2.696–14.843)	**<0.001**
ALBI > 1	1.423 (1.045–1.939)	0.025	1.291 (0.913–1.825)	0.148
AFP > 400 ng/mL	1.500 (1.098–2.048)	0.011	1.464 (1.046–2.049)	**0.026**
ITB > 50%	1.580 (1.140–2.191)	0.006	1.614 (1.130–2.307)	**0.009**
ECOG-PS > 0	1.587 (1.133–2.222)	0.007	1.311 (0.905–1.898)	0.152
MVI	1.963 (1.434–2.686)	<0.001	1.770 (1.251–2.503)	**0.001**
DAEs	0.780 (0.584–1.042)	0.093	0.755 (0.573–0.994)	**0.045**
Lymph nodes	1.411 (1.054–1.890)	0.021	1.645 (1.078–2.509)	**0.021**
Lungs	1.431 (1.018–2.013)	0.039	2.182 (1.331–3.578)	**0.002**
Bone	0.663 (0.440–0.998)	0.049	1.018 (0.586–1.766)	0.950
Adrenal glands	0.727 (0.434–1.218)	0.226		

AFP: alfa-fetoprotein; DAEs: dermatologic adverse events; ECOG-PS: Eastern Cooperative Oncology Group—Performance Status; ITB > 50%: intrahepatic tumor burden > 50%; MVI: macrovascular invasion. N.A. not applicable.

## Data Availability

The data presented in this study are available on request from the corresponding author. The data are not publicly available due to privacy restrictions.
